# Hepatotoxic Compounds and Mechanisms of Polygonum Multiflorum: A Narrative Review of Recent Advances

**DOI:** 10.3390/ijms27114733

**Published:** 2026-05-25

**Authors:** Yupeng Wang, Tianqi Ren, Yikun Zhang, Liyong Yuan, Xingchao Geng

**Affiliations:** 1 NHC Key Laboratory of Research on Quality and Standardization of Biotech Products and NMPA Key Laboratory for Quality Research and Evaluation of Biological Products, Institute for Biological Product Control, National Institutes for Food and Drug Control, Beijing 102629, China; wangyp@nifdc.org.cn; 2 National Center for Safety Evaluation of Drugs, National Institutes for Food and Drug Control, Beijing 100176, China; 3323092134@stu.cpu.edu.cn (T.R.); zhangyikun0522@163.com (Y.Z.)

**Keywords:** polygonum multiflorum, hepatotoxicity, components, molecular mechanisms, processing-based toxicity reduction

## Abstract

The clinical application of Polygonum multiflorum Thunb. (PM), a widely used traditional Chinese medicine, is increasingly constrained by its idiosyncratic hepatotoxicity. However, the precise chemical material basis (i.e., specific compound classes such as free anthraquinones, stilbene glycosides, and dianthrones) of this toxicity and the underlying synergistic mechanisms remain poorly defined, posing a significant challenge to safety assessment. This review systematically synthesizes the latest research progress over the past five years, aiming to elucidate the multi-component synergistic toxicity network of PM. As of November 2025, over 293 compounds have been characterized from PM, including anthraquinones, stilbene glycosides, and dianthrones. Among these, multiple components—particularly free anthraquinones (e.g., emodin, chrysophanol, physcion), cis-stilbene glycosides, and dianthrones—have been experimentally associated with hepatotoxicity in various in vitro and in vivo studies. However, current evidence is largely derived from in vitro or animal studies, and the quantitative nature of these synergistic interactions, as well as their translation to human clinical settings, remains uncertain. Accumulating evidence indicates that its hepatotoxicity does not originate from a single component but rather results from the synergistic interaction of free anthraquinones, stilbene glycosides, and dianthrones. At the mechanistic level, the toxicity involves a complex network encompassing direct cellular damage, an immune-mediated “triple-hit” cascade, and disruption of bile acid homeostasis. Importantly, traditional processing methods mitigate toxicity by reducing the content of specific toxic components, whereas individual genetic susceptibility (e.g., HLA-B*35:01 allele) appears to be an important contributing factor, although population-based quantitative risk estimates are still lacking in the occurrence of idiosyncratic liver injury. In conclusion, the hepatotoxicity of PM constitutes a multi-component, multi-target, and multi-pathway synergistic network. Future research should prioritize quantifying the toxic contribution of individual components and elucidating the quantitative principles governing their synergy, thereby establishing a robust paradigm for risk assessment.

## 1. Introduction

Polygonum multiflorum Thunb. (PM), the dried root tuber of Fallopia multiflora, is a traditional Chinese medicine widely utilized for purposes such as anti-aging and hair-darkening. Its chemical constituents include stilbene glycosides, anthraquinones, dianthrones, and other classes (flavonoids, tannins, etc.); among these, stilbene glycosides, free anthraquinones, and dianthrones are the most relevant to hepatotoxicity. Various analytical techniques (e.g., high-resolution mass spectrometry and multidimensional chromatography) have been developed to identify and quantify PM constituents [1,2,3]. Quantitative methods are also available for known key components [4,5,6,7]. Chemometric tools like Principal Component Analysis (PCA) and Orthogonal Partial Least Squares-Discriminant Analysis (OPLS-DA) are used to analyze the overall chemical differences between samples from different geographical origins or processing methods and to screen for key differential markers [2,8]. These tools help identify potential toxicity-related markers by correlating chemical profiles with toxicological outcomes. Current analysis of PM’s toxic components no longer relies on a single technology but has evolved into an integrated strategy combining efficient separation, high-resolution identification, accurate quantification, and intelligent data analysis. This technological system has significantly advanced the understanding of the complex chemical profile of PM. By 2020, over 245 chemical constituents had been discovered [9]. In the past five years, 48 new compounds have been isolated and structurally identified. Most of these new compounds remain stilbene glycosides and anthraquinones, such as Rumejaposide D, Chrysophanol anthrone, and Polydatin [3]. However, the toxicological relevance of these newly isolated compounds remains largely unexplored, as most have not been tested for hepatotoxic activity.

The processing of PM is a procedure involving heat treatment (with or without adjuvants) that induces complex chemical transformations, including hydrolysis, degradation, isomerization, and Maillard reactions. The main processing methods include steaming with black bean juice, clear steaming, processing with wine, the “nine-steaming nine-sun-drying” method, and others. Studies have found that different processing methods affect the chemical composition and toxicity of PM [1,4,10,11,12]. Although the Chinese Pharmacopoeia specifies the general processing method (e.g., stewing or steaming with black bean juice), it does not provide uniformly validated parameters for critical process variables such as duration, temperature, and number of cycles. Consequently, there is no definitive standard to determine whether PM has been adequately processed across different production practices. Despite the variety of methods, several key variables in the processing technique—such as duration, number of cycles, heating intensity, and the type and quantity of adjuvants—affect the final product quality and represent current challenges in research and standardization.

Although some controversy exists, most previous studies suggest that anthraquinones, stilbene glycosides, and dianthrones are the primary causes of liver injury [9,13]. Previously proposed mechanisms include (i) disruption of oxidative phosphorylation and the tricarboxylic acid cycle; (ii) interference with various metabolic pathways; and (iii) impairment of bile acid excretion. Due to the idiosyncratic nature of PM-induced hepatotoxicity, its detailed mechanisms remain incompletely understood. This review synthesizes recent advances with a focus on multi-component synergy and immune-mediated pathways. For this review, we searched SCOPUS, PubMed, and ScienceDirect for articles published between January 2020 and November 2025 using a combination of terms and phrases such as ‘Fallopia multiflora’, ‘Polygonum multiflorum’, ‘Fo-Ti’, ‘FoTi’, ‘liver injury’, ‘newly isolated compounds’, and ‘mechanism of hepatotoxicity’. Inclusion criteria were original research or review articles in English focusing on PM chemistry, hepatotoxicity, or processing. Exclusion criteria were studies on nonhepatic toxicities, conference abstracts, and duplicate reports.

## 2. Newly Isolated Compounds from PM (2021–2025)

As of 2020, more than 245 compounds have been isolated from PM, with stilbene glycosides and anthraquinones being the predominant constituents [9,14]. The present review focuses on the past five years (2021–2025). During this period, a systematic investigation by Wang et al. using UHPLC-Q-Exactive Orbitrap-MS led to the isolation and structural identification of 48 new compounds from processed PM. These include 9 anthraquinones, 7 stilbene glycosides, 5 flavonoids, 10 phenolic acids, and 17 other compounds (see Table 1) [3].

It should be noted that dianthrones, which are now recognized as potent hepatotoxic markers (discussed in Section 3.3 and Section 4), were already isolated and characterized before 2021; therefore, they are not listed in Table 1. Readers are referred to the comprehensive reviews by Lin et al. and Teka et al. for a complete compilation of hepatotoxic compounds isolated prior to 2021 [9,14].

For most of the newly isolated compounds listed in Table 1, toxicological data are not yet available. Where such information exists (e.g., polygonumnolides C1–C4, which showed weak hepatotoxicity on L-02 cells with IC_50_ values of 313.05, 205.20, 294.20, and 207.35 μM, respectively), it is indicated in the last column. For the majority, the activity column states “not tested”, reflecting the current knowledge gap.

## 3. The Material Basis of PM-Induced Hepatotoxicity

### 3.1. Anthraquinone Components

Anthraquinones have long been considered the primary source of hepatotoxicity in PM, although conflicting evidence exists (e.g., emodin also shows hepatoprotective effects at low doses), with their toxicity related to their existing forms. Free anthraquinones, including emodin, chrysophanol, and physcion, have been confirmed to possess significant direct hepatotoxicity [15]. In the study by Kang et al. found that exposing hepatocytes to emodin, chrysophanol, physcion, or other mixed anthraquinones at concentrations of 1, 5, 25, and 50 µM led to the accumulation of toxic bile acids by inhibiting bile acid transporter function and regulating their expression and related enzyme activities [16,17]. These findings suggest that free anthraquinones can disrupt bile acid homeostasis. Wang et al. discovered that emodin can covalently bind to cysteine residues of liver proteins, forming protein adducts, a direct molecular event triggering hepatocyte damage [18]. Notably, this covalent binding and associated hepatotoxicity were significantly enhanced under conditions of LPS- or BSO-induced oxidative stress, indicating that physiological state importantly influences toxic manifestation. Cytotoxicokinetic studies using HPLC-MS revealed that emodin exhibits time-dependent intracellular accumulation in the human hepatocyte line L-02, and its hepatocytotoxicity is significantly stronger than that of TSG and physcion [19]. Other studies have reported that emodin exhibits strong toxicity to zebrafish embryos [15]. However, some research also indicates that emodin shows a dose-dependent hepatoprotective effect against acetaminophen-induced acute liver injury in rats, contrasting with the aforementioned conclusions. This is consistent with previous findings of the dual effects of emodin. The dual role of emodin appears to be dose- and duration-dependent. A recent meta-analysis suggests a threshold of >45.74 mg/kg/day or >30.41 days for the switch from protection to toxicity. However, these values are derived from animal models and may not directly translate to humans [20]. Conjugated anthraquinones, such as emodin-8-O-β-D-glucoside (EG), have lower intrinsic toxicity but can be hydrolyzed by gut microbiota to free emodin, leading to delayed toxicity and individual variability [21]. This metabolic transformation process explains the time-dependent nature and individual differences in PM’s hepatotoxicity, which is closely related to gut microbiota composition. Furthermore, studies found that in the context of PM’s complex mixture, the absorption and distribution behavior of emodin change significantly, with its area under the curve (AUC) and peak concentration (Cmax) differing greatly from when administered alone [21]. This provides pharmacokinetic evidence for understanding why the overall toxicity of PM is greater than that of its individual components. Han et al. observed hepatotoxicity in zebrafish larvae treated with Emodin-8-O-β-D-glucopyranoside (Em8G), which was greater in males than in females. Transcriptomics combined with metabolomics showed that Em8G primarily affected carbohydrate metabolism (e.g., the TCA cycle) in male zebrafish and amino acid metabolism (e.g., arginine and proline metabolism) in females, suggesting that differences in energy metabolism impairment may be the underlying mechanism for Em8G-induced hepatotoxicity differing between sexes [22].

### 3.2. Stilbene Glycoside Components

Stilbene glycosides, particularly their stereoisomers, play a key role in the idiosyncratic liver injury induced by PM. Notably, the hepatotoxicity of cis-stilbene glucoside (cis-SG) is much greater than that of its trans isomer [23]. In an LPS-sensitized rat model of idiosyncratic liver injury, Meng et al. demonstrated that cis-SG at 50 mg/kg (but not trans-SG at the same dose) induced severe hepatic damage, including elevated plasma transaminases, inflammatory cytokine release, and hepatocyte apoptosis. This stark quantitative difference provides an important clue for understanding individual susceptibility to PM-induced liver injury [24]. Research by Meng et al. first elucidated the immunotoxic mechanism of cis-SG: in an LPS-sensitized rat model, cis-SG inhibited the PPAR-γ pathway, leading to overactivation of the NF-κB signaling pathway, triggering the release of large amounts of pro-inflammatory cytokines (such as TNF-α, IL-6, and IL-1β) and causing severe immune-mediated liver damage [24]. This pathway has since been confirmed by another independent study [25]. Importantly, pretreatment with the PPAR-γ agonist pioglitazone effectively reversed this damage, providing a potential target for clinical intervention. The toxicity mechanism of stilbene glycosides also involves a complex metabolic activation process. Pan et al.’s study revealed a two-step metabolic activation mechanism for TSG: first, hydrolysis by intestinal β-glucosidase to its aglycone, followed by oxidation by hepatic CYP3A4/CYP2C9 to a highly reactive ortho-benzoquinone intermediate. This intermediate can act as a hapten, covalently binding to bodily proteins to form complete antigens, thereby triggering a specific immune response [5]. Although this two-step activation has been demonstrated in mouse models and human liver microsomes, the quantitative contribution of this pathway in humans, particularly the interindividual variability due to gut microbiota composition, requires further investigation.

### 3.3. Dianthrone Components

In the past five years, dianthrone components have been investigated in at least 9 independent studies due to their potent cytotoxicity. Ma’s research group, through a series of studies, has confirmed the hepatotoxic effects of dianthrones [4,7,11,15]. In 2020, while screening PM for hepatotoxic components using a zebrafish larvae model, they discovered that dianthrone compounds (such as trans-emodin dianthrone and cis-emodin dianthrone) exhibited significant hepatotoxicity [15]. In 2021, using UHPLC-QQQ-MS/MS technology, they precisely determined the content of dianthrones in PM and confirmed on a HepaRG cell model that trans-emodin dianthrone and cis-emodin dianthrone had very low IC_50_ values (10.98 µM and 15.45 µM, respectively), exhibiting cytotoxicity far stronger than monomeric anthraquinones [7]. However, direct human pharmacokinetic data for dianthrones are lacking. A rat pharmacokinetic study reported low oral bioavailability (2.83%) and relatively low plasma concentrations for trans-emodin dianthrone, indicating that direct extrapolation from in vitro IC_50_ to clinical risk is not straightforward [26]. Whether the in vitro cytotoxic concentrations are achievable in the human liver remains unknown, and further human pharmacokinetic studies are needed. In 2022, they systematically assessed changes in dianthrone content under different processing methods and found a significant correlation between dianthrone content in PM and hepatotoxicity, particularly for trans-emodin dianthrone and cis-emodin dianthrone [11]. Furthermore, Wang et al.’s study on the structure–activity relationship of trans-emodin dianthrone and cis-emodin dianthrone found that their inhibitory capacity (Ki values of 0.863 µM and 1.083 µM, respectively) against UGT1A1, a key enzyme in bilirubin metabolism, was stronger than that of emodin, which might be an important reason for PM-induced cholestatic liver injury [27]. Additionally, Li et al. found that trans-emodin dianthrone exhibited significant hepatotoxicity at the HepG2 cellular level, with mechanisms related to the inhibition of the antioxidant system via the JNK/Bax and PI3K/AKT/mTOR pathways, causing mitochondrial dysfunction and inducing apoptosis [28].

### 3.4. Other Potential Toxic Components

It should be emphasized that the potential toxic components described below are based on preliminary or computational evidence and require experimental validation. Beyond the three main categories mentioned above, research suggests other components may participate in PM’s hepatotoxicity. Tannins were reported in early studies to have hepatotoxicity, possibly related to protein-binding precipitation [13]. Network toxicology analyses predict that flavonoid components like quercetin and kaempferol may also be involved by acting on multiple hepatotoxicity targets [29]. Furthermore, phospholipid components might act as carriers, indirectly participating in cholestatic liver injury by altering the physicochemical properties of bile [13]. While the contribution of these “minor components” requires further validation, they suggest that the material basis of PM’s hepatotoxicity may be more complex than currently understood.

### 3.5. The Complex and Synergistic Network of Multi-Component Interaction

Current research posits that the hepatotoxicity of PM is not a simple additive effect of single components but rather a complex synergistic network. However, most synergy studies rely on fixed-ratio combinations in vitro, which may not reflect the dynamic, variable exposure in vivo. Moreover, the lack of standardized synergy metrics (e.g., combination index) across studies hampers direct comparison. A well-documented example of synergy involves the interaction between TSG and emodin. TSG significantly inhibits the metabolism of emodin by suppressing the expression of UGT1A8, UGT1A10, UGT2B7, CYP3A4, and CYP2C19 in cells, while also promoting its absorption. This leads to a 1.96-fold increase in emodin’s systemic exposure and a 1.66-fold increase in liver protein adduct levels, producing a significant synergistic toxicity-enhancing effect [30,31]. More complexly, Deng et al., using an innovative immune-liver-on-a-chip (i-LOC) system, found that even when known toxic components (cis-SG, EG, etc.) were combined in proportion, their toxicity remained far lower than that of the total PM extract [32]. The i-LOC system represents an innovative microphysiological model, but it has only been reported by a single research group. Its reproducibility across laboratories and correlation with human in vivo responses have not yet been established.

Several studies have reported seemingly contradictory findings regarding interactions among PM components. Yu et al. found that TSG and physcion might attenuate the hepatotoxicity of emodin, although the detoxification mechanism remains unclear [33]. In contrast, Wang et al. reported that EG is a protoxin that is metabolically activated to emodin and that the processing-induced reduction in EG content is a key detoxification mechanism [34]. Zhang et al. further demonstrated that EG induces liver injury by disrupting sphingolipid and bile acid metabolic pathways and that TSG significantly intensifies this damage [35]. These contradictory findings may be explained by differences in experimental models (e.g., cell lines vs. animals), dosage ratios, and exposure duration. For instance, TSG may enhance emodin-induced toxicity at high ratios but attenuate it at low ratios through antioxidant effects. Future studies should systematically investigate dose–response and ratio–response surfaces to resolve these discrepancies.

These findings present new challenges for the safety evaluation of PM, indicating that research strategies based on single components or limited combinations may have limitations.

## 4. Impact of Processing on the Chemical Components of PM

Processing is a crucial technique in traditional Chinese medicine theory for “reducing toxicity and enhancing efficacy.” For PM, this process is particularly critical. Recent research utilizing modern analytical techniques, such as multidimensional chromatography coupled with high-resolution mass spectrometry, has profoundly revealed the complex effects of processing on PM’s chemical composition. The core changes are first reflected in the significant reduction in toxic components. Studies confirm that dianthrone components in PM are potential hepatotoxicity markers. During processing methods like the “nine-steaming nine-sun-drying” method, the content of these components decreases sharply with increasing processing cycles, even falling below the detection limit, which closely correlates with the significantly reduced hepatotoxicity observed in in vivo experiments [4]. Using an online two-dimensional liquid chromatography–mass spectrometry approach, Fan et al. systematically characterized 150 compounds from raw and processed PM and quantified 12 differential components. They found that potentially toxic compounds such as cis-2,3,5,4′-tetrahydroxystilbene-2-O-β-D-glucoside (cis-THSG) and emodin-8-O-β-D-glucoside gradually decreased during the nine cycles of steaming and sunning, irrespective of the addition of black beans [2].

Free anthraquinones (such as emodin and chrysophanol) exhibit a more complex change trend: in the early stages of processing, their content may increase due to the hydrolysis of conjugated anthraquinone glycosides, accompanied by spatial redistribution within the plant tissue [1]. However, excessive processing may lead to their degradation. Mass spectrometry imaging visually demonstrates this dynamic process. Although free anthraquinones themselves are toxic, processing overall still demonstrates toxicity reduction. A direct comparison of raw vs. processed PM in zebrafish and mice confirmed that processed formulations exhibit significantly reduced hepatotoxicity compared to their raw counterparts.

In addition to direct toxicity reduction, processing also induces profound transformations in PM’s pharmacologically active components. The most notable is the substantial decrease in the content of stilbene glycosides, particularly 2,3,5,4′-tetrahydroxystilbene-2-O-β-D-glucoside (TSG), after processing. Du et al. systematically analyzed the effect of steaming on PM’s constituents, defecation, and liver injury. They found that raw PM promoted defecation, whereas the steamed product (PMP) did not. Only a high dose of raw PM administered for 14 days caused mild liver injury, which disappeared after 14 days of drug withdrawal. Using network pharmacology and molecular docking, the authors predicted that TSG, emodin, and physcion are the most effective constituents in promoting defecation and causing liver injury. The reduction in TSG content after steaming correlates with the diminished laxative effect and lower hepatotoxicity of the processed product [12].

On the other hand, processes such as hydrolysis and Maillard reactions during processing lead to the generation of new compounds, such as 5-hydroxymethylfurfural (5-HMF) and succinic acid. Simultaneously, the content of phenolic acids (e.g., gallic acid) and flavonoids (e.g., catechin) may also increase [2]. When black beans are used as adjuvants, processing introduces exogenous active components such as soybean isoflavones [2]. Furthermore, processing also affects PM polysaccharides, shifting their molecular weight distribution towards lower molecular weights, which may be related to the adjustment of their pharmacological activity [2].

These changes in chemical components ultimately collectively lead to the transformation of PM’s pharmacological and toxicological properties. An important indirect detoxification mechanism is manifested in the weakening of inhibitory effects on drug-metabolizing enzymes. Research has found that raw PM strongly inhibits the cytochrome P450 enzyme system (especially CYP1A2), whereas after processing, particularly with the “nine-steaming nine-sun-drying” product, this inhibitory effect is significantly reduced. This is primarily attributed to the decreased content of key inhibitory components, such as gallic acid, during processing, thereby substantially lowering the risk of PM interacting with other drugs [10]. Clinically, this CYP inhibition could increase the plasma concentration of co-administered drugs metabolized by CYP1A2/CYP3A4 (e.g., theophylline, midazolam), potentially leading to adverse drug–drug interactions. This risk has been experimentally validated; for instance, Xing et al. demonstrated in rats that co-administration of raw PM increased the systemic exposure of the probe drugs sulindac and psoralen, consistent with the inhibition of their respective metabolic enzymes. These findings underscore the clinical relevance of CYP-mediated herb–drug interactions and highlight the necessity for clinical pharmacokinetic studies to guide the safe use of PM [10].

In summary, processing achieves precise regulation of PM’s components through a multidimensional and dynamic chemical restructuring process. The core of its “toxicity reduction” mechanism lies in the efficient removal of highly toxic dianthrones and the transformation and modification of components such as free anthraquinones and stilbene glycosides, along with the introduction of beneficial exogenous components, reshaping PM’s chemical profile. This not only directly reduces inherent toxicity but also lowers potential medication risks by attenuating the inhibition of metabolic enzymes. However, there is still a lack of unified standards for determining the optimal processing endpoint. Future research needs to establish more precise quality control systems and deeply elucidate the biological effects of newly generated components during processing to provide a solid foundation for the safe and effective clinical application of PM.

## 5. Molecular Mechanisms of PM-Induced Hepatotoxicity

**Figure 1:**
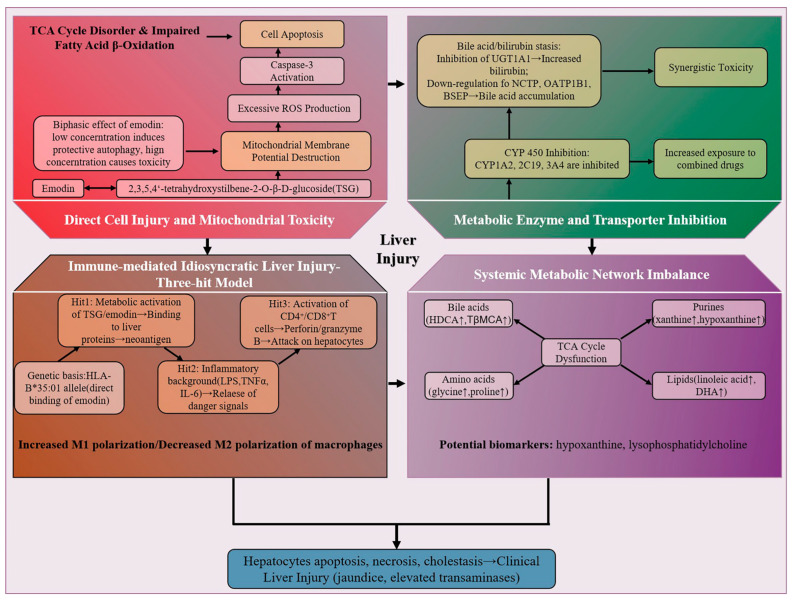
Schematic diagram of the molecular mechanism of Polygonum multiflorum-induced hepatotoxicity.

The mechanisms underlying the hepatotoxicity of PM are complex, involving direct cellular damage, immune-mediated idiosyncratic reactions, systemic metabolic disturbances, and interference with drug-metabolizing enzyme systems. These processes are interconnected, collectively forming a complex toxicological network (Figure 1).

Current research widely considers mitochondrial toxicity to be a core event in direct cellular damage. Anthraquinone components in PM, such as emodin, can disrupt mitochondrial membrane potential in a concentration- and time-dependent manner, leading to dysfunction, subsequent massive production of reactive oxygen species (ROS), and caspase-3-mediated apoptosis [35]. While mitochondrial toxicity is widely considered a core event, alternative or parallel mechanisms exist, such as endoplasmic reticulum stress and direct activation of death receptors [36]. These pathways may also contribute independently. Metabolomic studies provide systematic evidence at the systems level, revealing significant alterations in TCA cycle intermediates and acylcarnitine profiles in rats with liver injury, indicating severe impairment of mitochondrial fatty acid β-oxidation and energy metabolism [37,38,39]. These metabolic alterations are not only a consequence of toxicity but may also exacerbate damage through positive feedback loops. Notably, certain components like emodin exhibit biphasic effects: at low concentrations, they may induce protective autophagy by activating the PI3K/AKT/mTOR pathway, while switching to inhibition at high concentrations, revealing the complexity of their toxicity [40]. The biphasic effect—low-dose autophagy activation vs. high-dose apoptosis—has been observed primarily in hepatocyte cell lines. The switch point varies with cell type and culture conditions, and it remains unknown whether such a biphasic response occurs in human liver in vivo. Moreover, the therapeutic window is likely very narrow.

For the clinically unpredictable IDILI, we propose a hypothetical “three-hit” model to provide a comprehensive explanatory framework. This model is conceptual and based on indirect evidence from animal studies and in vitro experiments. Direct proof in humans is still lacking. The model posits that the first hit originates from reactive intermediates formed during the metabolic activation of PM components such as TSG and emodin. These intermediates covalently bind to liver proteins, forming neoantigens [5,18,41]. The second hit is provided by a background of mild inflammation caused by factors such as infection or fatigue, which releases “danger signals” like LPS and inflammatory cytokines (TNF-α, IL-6), thereby activating liver immune cells [24]. The third hit involves the aberrant activation of adaptive immunity, where neoantigens are presented within the inflammatory microenvironment, leading to the activation of CD4+ and CD8+ T cells. These cells then attack hepatocytes via mechanisms such as perforin/granzyme B pathways [24,25]. Future studies using human liver organoids or clinical samples are needed to validate this model. Macrophage polarization also plays a crucial role. PM disrupts immune homeostasis by modulating sphingolipid and JAK-STAT signaling pathways, promoting the polarization of pro-inflammatory M1 macrophages while suppressing anti-inflammatory M2 macrophages [42,43]. Crucially, genetic susceptibility forms the foundation of this model. The HLA-B*35:01 allele has been identified as a significant risk factor. In a landmark case–control study, the frequency of HLA-B*35:01 was 45.4% in PM-DILI patients compared with 2.7% in the general Han Chinese population (odds ratio [OR] = 30.4, 95% CI: 11.7–77.8) [44]. This finding underscores a strong genetic predisposition, although its positive predictive value remains limited due to the rarity of DILI, indicating that other factors are also required. Furthermore, Zeng et al.’s research revealed that emodin can directly bind to the product of this allele, activating CD8+ T cells through a “pharmacological–immune interaction” mechanism, providing a direct immunobiological explanation for individual differences [44].

The broad inhibition of drug-metabolizing enzyme systems by PM is an important mechanism underlying drug–drug interactions. Studies have found that raw PM significantly inhibits various CYP450 enzymes, especially CYP1A2. Moreover, TSG exhibits mechanism-based irreversible inhibition of CYP2C19 and CYP3A4, substantially increasing the exposure risk of co-administered drugs [10,30]. All current evidence comes from in vitro enzyme assays or animal pharmacokinetics. Clinical validation in human subjects is urgently needed, as extrapolation from in vitro data often overestimates the actual degree of inhibition due to protein binding and intestinal metabolism. Concurrently, the synergistic inhibition of bilirubin and bile acid metabolic pathways is central to PM-induced jaundice and cholestasis. On one hand, multiple anthraquinone components (e.g., dianthrones) potently inhibit UGT1A1, the key enzyme for bilirubin conjugation. On the other hand, PM and its main components, TSG and EG, synergistically downregulate the expression of hepatic uptake transporters (NTCP, OATP1B1/3) and efflux transporters (MRP2, BSEP) while upregulating MRP3. This leads to the intrahepatic accumulation of bile acids and bilirubin, resulting in synergistic toxicity [27,45].

Metabolomic studies reveal that PM-induced hepatotoxicity is accompanied by a profound disruption of systemic metabolic homeostasis. Bile acid metabolism dysregulation is a prominent feature, with serum hyodeoxycholic acid (HDCA) and urinary tauro-β-muricholic acid (TβMCA) identified as potential biomarkers [46]. More extensive disturbances across metabolic networks include imbalances in amino acid metabolism (e.g., elevated levels of glycine, proline, and alanine), lipid metabolism (e.g., increased levels of linoleic acid, DHA, and cholesterol, suggesting accelerated lipid peroxidation), and energy metabolism (abnormalities in intermediates like lactate and citrate). The functional impairment of the TCA cycle is a central hub for these disturbances [47]. Additionally, aberrations in purine metabolism (elevated xanthine, hypoxanthine) and glycerophospholipid metabolism have been confirmed to participate. Studies based on clinical samples have further screened hypoxanthine, lysophosphatidylcholine, and others as potential high-efficiency diagnostic biomarkers [48,49].

The latest systems biology research, such as integrating network toxicology with spatially resolved metabolomics, indicates that PM hepatotoxicity is a network event involving the crosstalk of multiple pathways. Studies confirm the central roles of signaling pathways like PI3K-Akt, AMPK, MAPK, and mTOR. Molecular docking has validated the high binding affinity of toxic components to key targets such as mTOR and AKT1 [50]. Spatial metabolic phenotyping visually correlates the abnormal distribution of metabolites (e.g., taurine, acylcarnitines) in the liver with tissue damage. Pathway enrichment analysis shows that pathways like linolenic acid metabolism and carnitine synthesis collectively point to four core pathological phenotypes: cholestasis, mitochondrial damage, oxidative stress, and energy metabolism disorder [50].

In summary, PM-induced hepatotoxicity begins with the direct damage to hepatocytes by toxic components and the inhibition of key metabolic enzymes/transporters, triggering systemic metabolic disturbances. In genetically susceptible individuals, these initial events can activate a specific immune attack through the “pharmacological–immune interaction” mechanism and the “three-hit” model, ultimately leading to clinical liver injury.

## 6. Summary and Future Perspectives

### 6.1. Research Summary

Research on the hepatotoxicity of PM has progressed from the stage of “phenomenological observation” to mechanistic elucidation. Three major shifts have occurred: (1) from single-component thinking to a synergistic network perspective, with dianthrones and cis-stilbene glycosides newly recognized as key toxic contributors; (2) from direct cytotoxicity to immune-mediated mechanisms, exemplified by the ‘three-hit’ hypothesis; and (3) from qualitative description to quantitative and engineering approaches, including organ-on-a-chip and machine learning. Regarding the material basis, the focus has expanded from anthraquinones alone to a complex spectrum comprising stilbene glycosides (particularly the cis-isomers) and dianthrones. Concerning toxic mechanisms, an integrated mechanistic model has been established, covering direct cellular damage, abnormal immune activation, and metabolic network disturbances. Notably, the “three-hit” model provides a novel framework for understanding IDILI. In terms of research technologies, the application of new techniques such as metabolomics, i-LOC, and zebrafish models has significantly advanced the field towards systematization and refinement. Regarding processing principles, the detoxification essence of traditional processing methods has been scientifically elucidated from both the perspectives of chemical component transformation and biological effects.

### 6.2. Future Perspectives

Despite significant progress, research on PM hepatotoxicity still faces numerous challenges. First, the mechanisms underlying individual susceptibility require more in-depth exploration. Although the HLA-B*35:01 allele has been associated with PM-induced liver injury, the specific mechanisms of immune recognition and response remain unclear. Future studies need to integrate organoid models and immune cell profiling technologies to deeply analyze how genetic polymorphisms determine individual susceptibility. Second, the construction of a precise toxicological evaluation system is an important future direction. It is necessary to integrate chemical fingerprints, biological effects, and individual genomic information to establish predictive models for PM hepatotoxicity. Discovered biomarkers should be advanced to clinical validation for early warning. Simultaneously, greater efforts are needed to verify the consistency between basic research findings and clinical realities. Third, processing techniques require precision and standardization. Based on the patterns of chemical changes and toxicity fluctuations, standards for determining the processing endpoint should be established using the content of toxic components as indicators, aiming to modernize processing techniques and achieve precise quality control. Existing guidelines, such as the Chinese Pharmacopoeia (2025 edition) and WHO guidelines on herbal medicine quality control, provide a starting point but do not specify validated endpoints for PM. Future efforts should harmonize methods across research groups and regulatory bodies. It is crucial to resolve controversies among different research groups regarding optimal processing parameters and establish unified procedural standards. Finally, quantitative research on multi-component interactions urgently needs methodological innovation. For instance, introducing mathematical modeling and artificial intelligence technologies could more accurately decipher the interaction patterns among the multiple components in PM, thereby providing new research pathways and methods for the toxicity assessment of complex traditional Chinese medicine systems. Although still exploratory, mathematical modeling and machine learning have already been applied to predict the toxicity of herbal mixtures (e.g., using quantitative structure–activity relationship models). Future research should focus on integrating multi-omics data to build predictive models with external validation.

## Figures and Tables

**Table 1:** Recently isolated new compounds from PM reported between January 2020 and November 2025.

No.	Compound Name	Class of Compound	Source	Molecular Formula
1	Rumejaposide D	Anthraquinones	PMRPW, PMRPE	C_21_H_22_O_11_
2	Di-emodin-Di-glucoside	Anthraquinones	PMRPW	C_42_H_42_O_18_
3	Citreorosein-O-glucoside	Anthraquinones	PMRPW, PMRPE	C_21_H_20_O_11_
4	Emodin-O-glucoside- gallate	Anthraquinones	PMRPE	C_28_H_24_O_15_
5	6-Carboxyl emodin	Anthraquinones	PMRPW, PMRPE	C_16_H_10_O_7_
6	Chrysophanol anthrone	Anthraquinones	PMRPE	C_15_H_12_O_3_
7	Hydroxyl-rhein	Anthraquinones	PMRPW, PMRPE	C_15_H_8_O_7_
8	Digitolutein	Anthraquinones	PMRPE	C_16_H_12_O_4_
9	Emodin anthrone	Anthraquinones	PMRPW, PMRPE	C_15_H_12_O_4_
10	Rhapontin	Stilbenes	PMRPW, PMRPE	C_21_H_24_O_9_
11	β-D-glucoside,4-[2,3- dihydro-3-(hydroxymethyl)- 5-(3-hydroxypropyl)-7- methoxy-2-yl]-2- methoxyphenyl	Stilbenes	PMRPW, PMRPE	C_26_H_34_O_11_
12	Resveratrol	Stilbenes	PMRPW, PMRPE	C_14_H_12_O_3_
13	Multiflorumisides A	Stilbenes	PMRPW, PMRPE	C_40_H_44_O_18_
14	Tetrahydroxystilbene-O- (caffeoyl)-glucoside	Stilbenes	PMRPE	C_29_H_28_O_12_
15	Polydatin	Stilbenes	PMRPW, PMRPE	C_20_H_22_O_8_
16	Isorhapontigenin	Stilbenes	PMRPW, PMRPE	C_15_H_14_O_4_
17	Acetyl-epicatechin-O- glucoside	Flavonoids	PMRPW, PMRPE	C_23_H_26_O_12_
18	Hesperetin-7-O-glucoside	Flavonoids	PMRPW, PMRPE	C_22_H_24_O_11_
19	Cirsimarin	Flavonoids	PMRPW, PMRPE	C_23_H_24_O_11_
20	Kaempferol	Flavonoids	PMRPW, PMRPE	C_15_H_10_O_6_
21	Kaempferol-O-glucoside- rhamnose	Flavonoids	PMRPW, PMRPE	C_27_H_30_O_15_
22	Dihydroxy-benzoic acid	Phenolic acids	PMRPW, PMRPE	C_7_H_6_O_4_
23	Galloyl-glycerol	Phenolic acids	PMRPW, PMRPE	C_10_H_12_O_7_
24	Vanillic acid	Phenolic acids	PMRPW, PMRPE	C_8_H_8_O_4_
25	Caffeic acid	Phenolic acids	PMRPW, PMRPE	C_9_H_8_O_4_
26	1-(5-Methylfuran-2-yl) ethanone	Phenolic acids	PMRPW, PMRPE	C_7_H_8_O_2_
27	Veratric acid	Phenolic acids	PMRPW, PMRPE	C_9_H_10_O_4_
28	Coumaric acid	Phenolic acids	PMRPW, PMRPE	C_9_H_8_O_3_
29	2-Methyl gallic acid	Phenolic acids	PMRPW, PMRPE	C_8_H_8_O_5_
30	Methyl gallate	Phenolic acids	PMRPW, PMRPE	C_8_H_8_O_4_
31	Syringic acid	Phenolic acids	PMRPE	C_9_H_10_O_5_
32	Citric acid	Others	PMRPW, PMRPE	C_6_H_8_O_7_
33	L-Tyrosine	Others	PMRPW, PMRPE	C_9_H_11_NO_3_
34	3-O-feruloylquinic acid	Others	PMRPW, PMRPE	C_17_H_20_O_9_
35	Altechromone A	Others	PMRPW, PMRPE	C_11_H_10_O_3_
36	Acetyl 1-methyl-1- acetoxyethyl peroxide	Others	PMRPW, PMRPE	C_7_H_12_O_5_
37	P-hydroxybenzal-dehyde	Others	PMRPW, PMRPE	C_7_H_6_O_2_
38	Vanillin	Others	PMRPW, PMRPE	C_8_H_8_O_3_
39	Nudiposide	Others	PMRPW, PMRPE	C_27_H_36_O_2_
40	(+)-lyoniresinol-2α-O-β- glucoside	Others	PMRPW, PMRPE	C_28_H_38_O_13_
41	Cinnamyl-galloyl-O- glucoside	Others	PMRPW, PMRPE	C_22_H_22_O_11_
42	2-Methyl-5-carboxymethyl-7-hydroxychromone	Others	PMRPE	C_12_H_10_O_5_
43	Trans-N-caffeoyltyramine	Others	PMRPW, PMRPE	C_17_H_17_NO_4_
44	Noreugenin	Others	PMRPW, PMRPE	C_10_H_8_O_4_
45	1,2-Dihydroxypropane-1-(4-hydroxy-phenyl)	Others	PMRPW, PMRPE	C_9_H_12_O_3_
46	Thunberginol C-6- O-β-D-glucoside	Others	PMRPW, PMRPE	C_21_H_22_O_10_
47	Torachrysone	Others	PMRPE	C_14_H_14_O_4_
48	3,8-Dihydroxy-1-methoxyxanthone	Others	PMRPW, PMRPE	C_14_H_10_O_5_

PMRPW: Polygoni Multiflori Radix Praeparata water extract; PMRPE: Polygoni Multiflori Radix Praeparata ethanol extract. None of the newly isolated compounds has been experimentally evaluated for hepatotoxicity to date, except where noted.

## Data Availability

No new data were created or analyzed in this study. Data sharing is not applicable to this article.
